# Supplementary figures and images for: Real-Time Monitoring of Lysosomal Membrane Permeabilization Using Acridine Orange
Source: Methods Protoc. 2023 Aug 9;6(4):72. doi: 10.3390/mps6040072 (PMC10459729; doi:10.3390/mps6040072)

## Western blots to Figure 5F

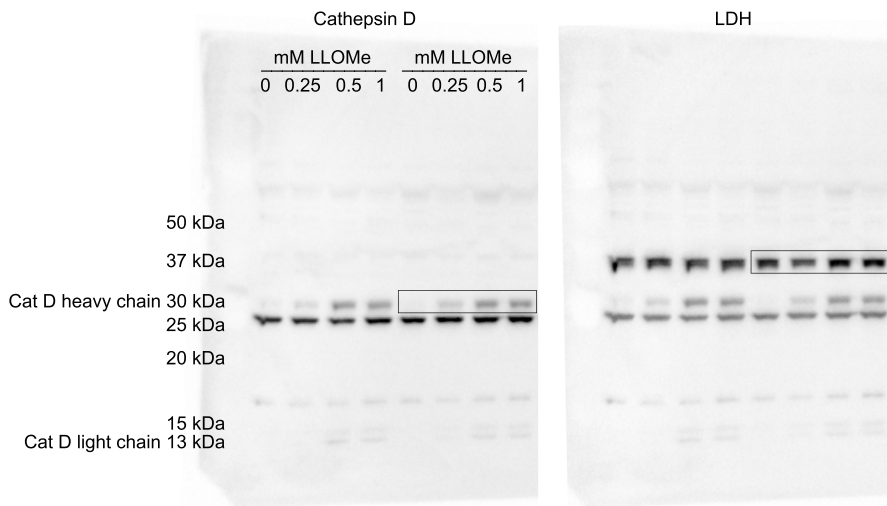

## Western blots to Figure 5G

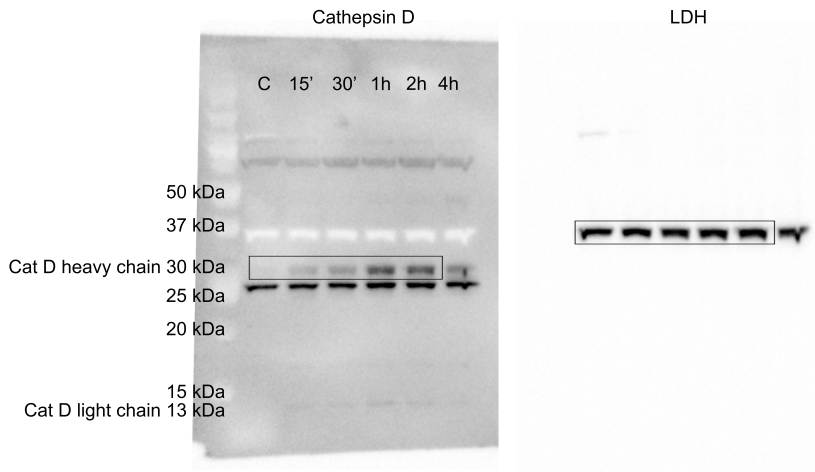

Supplement: Supplementary file 1 [file mps-06-00072-s001.zip › mps-2504567-supplementary.pdf]
